# Depression, risk factors, and coping strategies in the context of social dislocations resulting from the second wave of COVID-19 in Japan

**DOI:** 10.1186/s12888-021-03047-y

**Published:** 2021-01-12

**Authors:** Yuko Fukase, Kanako Ichikura, Hanako Murase, Hirokuni Tagaya

**Affiliations:** Kitasato University School of Allied Health Sciences, Kitazato 1-15-1, Minami-ku, Sagamihara, Kanagawa 252-0373 Japan

**Keywords:** The second wave of COVID-19, Mental health, Prevalence, General population, Risk factor for increased depression, Coping strategies

## Abstract

**Background:**

Social dislocations resulting from coronavirus disease 2019 (COVID-19) pandemic have been prolonged, which has led to general population social suppression. The present study aimed to reveal risk factors associated with mental health problems and suggest concrete coping strategies in the context of COVID-19.

**Methods:**

A web-based survey was conducted in July when Japan was experiencing a second wave of COVID-19. Demographics, Patient Health Questionnaire-9 (PHQ-9), state anger, anger control, and the Brief Coping Orientation to Problems Experienced were measured. Multivariate logistic regression analysis on PHQ-9 scores by set variables was conducted.

**Results:**

The participants were 2708 individuals, and 18.35% of them were depressed. Logistic regression analysis showed that in the order of odds ratios (ORs), underlying disease (OR = 1.96, 95% confidence interval (CI) = 1.32–2.92), not working (OR = 1.85, CI = 1.22–2.80), negative economic impact (OR = 1.33, CI = 1.01–1.77), state anger (OR = 1.17, CI = 1.14–1.21), anger control (OR = 1.08, CI = 1.04–1.13), age (OR = 0.97, CI = 0.96–0.98), high income (OR = 0.45, CI = 0.25–0.80), and being married (OR = 0.53, CI = 0.38–0.74) were predictors of depressive symptoms. Regarding coping strategies, planning (OR = 0.84, CI = 0.74–0.94), use of instrumental support (OR = 0.85, CI = 0.76–0.95), denial (OR = 0.88, CI = 0.77–0.99), behavioural disengagement (OR = 1.28, CI = 1.13–1.44), and self-blame (OR = 1.47, CI = 1.31–1.65) were associated with probable depression.

**Conclusions:**

During prolonged psychological distress caused by COVID-19 pandemic, the prevalence of depressive symptoms in Japan was two to nine times as high as before the COVID-19 pandemic, even though Japan was not a lockdown country. Although some coping strategies were useful for maintaining mental health, such as developing ways, alone or with others, to address or avoid social dislocations, the influence of demographics was more powerful than these coping strategies, and medical treatments are needed for high-risk individuals.

**Supplementary Information:**

The online version contains supplementary material available at 10.1186/s12888-021-03047-y.

## Background

The outbreak of severe acute respiratory syndrome coronavirus (SARS-CoV-2) infection and the resulting coronavirus disease 2019 (COVID-19) was first identified in Wuhan, China, in December 2019. The number of infected people continues to increase around the world [1], and there is no indication that the situation is being brought under control because a treatment for COVID-19 is under development and preventative measures are still obscure. The COVID-19 pandemic has caused social dislocations, that is, more than 100 countries had been locked down, and many other countries had restricted a person’s activities and movements to prevent the spread of infection [2].

Social dislocations influence mental health among the general population. Regarding lockdown countries, almost 17.6 to 48.3% of the general population reported depression in China [3–6], and over 71% of the general population showed psychological distress in Spain [7, 8]. Despite no lockdown in Hong Kong, 19% of the general population had depression, and 25.4% reported that their mental health had deteriorated [9].

Some risk factors for worsening mental health because of social dislocations have been revealed: being female [4, 8, 10–20], having a medical history, such as prior psychiatric illness, physical illness, or chronic disease [3, 10, 13, 15, 19], being unmarried [11, 21, 22], having lower income [3, 11, 23], and experiencing a negative economic impact [24].

In the context of social dislocations, particular coping strategies have been suggested as adaptive for maintaining mental health. Wang et al. [21] showed that a positive coping style was effective for psychological distress and that a negative coping style was ineffective. Li [25] showed that using both emotion- and problem-focused coping was better for psychiatric status, and using only problem-focused coping was associated with a high PTSD level. On the other hand, Guo et al. [24] showed that the use of problem-focused coping was associated with fewer mental health problems, while emotion-focused coping might increase mental health problems.

These differences might have been caused by the survey period. For example, problem-focused coping relieves psychological distress; however, when psychological distress is prolonged, problem-focused coping aggravates psychological distress because using problem-focused coping requires a clear sensorium [26–28]. As the social suppression caused by the COVID-19 pandemic will be needed for several months [29, 30], it is necessary to investigate coping strategies for prolonged psychological distress. Most importantly, coping strategies consist of several factors in addition to those that are positive/negative and problem-focused/emotion-focused [31]; however, the coping strategies examined in previous studies were too simple to use in daily life, and concrete coping strategies for social dislocations are needed.

**Fig. 1:**
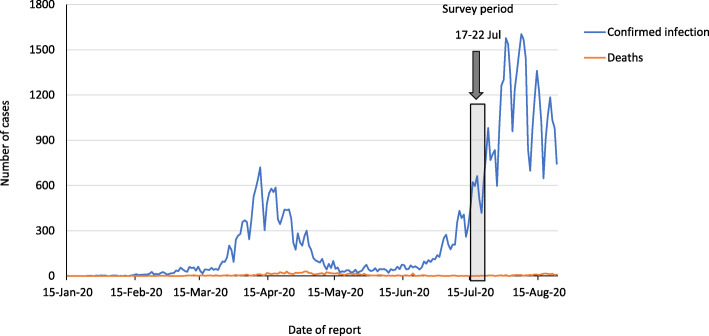
Number of confirmed and dead COVID-19 cases in Japan. Data from JX Press Corporation (2020) https://jxpress.net/

The present study aimed to identify the risk factors for mental health problems linked with prolonged psychological distress caused by the COVID-19 pandemic in Japan; the identification of these factors may also lead to the identification of adaptive and concrete coping strategies. In Japan, the first infected person was identified in January, and the number of infected persons and associated deaths sharply increased from late March to April (Fig. 1). In April, the government of Japan declared a state of emergency for all 47 prefectures. The state of emergency requested self-restraint. In the 13 prefectures where infection had spread significantly, the government advised against going outside and recommended self-restraint for an extended period of time. As the spread gradually subsided in May, the government lifted the state of emergency on May 25 throughout Japan. However, the number of infected persons gradually increased from the beginning of July, and the second wave of COVID-19 occurred in mid-July. Although Japan had no lockdown, the general population was asked to show self-restraint and maintain distance from other people. As this second wave of COVID-19 might disappoint and exhaust the general population, concrete coping strategies against long-term social dislocations are needed. Based on this information, in order to identify the prevalence of probable depression during the second wave of the COVID-19 pandemic in Japan, as well as to identify strong risk factors, and propose some coping strategies, a web based survey for the general population who have lived in the 13 prefectures was conducted in July 2020.

## Methods

### Survey design and recruited participants

We conducted a web-based survey between 17 and 22 July 2020 with an online research company, Macromill, Inc. Japan. The present study required more than 440 participants to be categorized into the probable depression group because there were 44 explanatory variables in the logistic regression analysis, as mentioned below, and the sample size needed to be 10 times the number of explanatory variables [32]. Based on a pervious study [3, 33], the prevalence of depressive symptoms during the COVID-19 pandemic may be assumed to be less than 17%. Therefore, the present study required 2700 participants. From a pool of approximately 10 million registered individuals residing in Macromill, Inc., we aimed to recruit 2700 participants who lived in the 13 prefectures under special precautions that were related to the COVID-19 pandemic: Tokyo, Saitama, Chiba, Kanagawa, Osaka, Hyogo, Fukuoka, Hokkaido, Ibaraki, Ishikawa, Gifu, Aichi, and Kyoto. The selection criteria were being 20 to 69 years old and a quota sampling method was used to compare equal-sized age groups (20s, 30s, 40s, 50s, 60s, and 70s), sex (male and female), and employment status (full-time worker; no regular employment; and unemployed, including homemaker, retired, and jobless). All the participants received Macromill points for their participation; Macromill points is the original point service of Macromill, Inc., and participants can trade these points for something prize and cash.

### Measures

Demographics: Age, sex, employment status, residential area, with or without an underlying disease that was associated with a higher risk of a more severe SARS-CoV-2 infection, marital status, household income, and economic impact of the COVID-19 pandemic. The underlying diseases with a higher risk of a more severe infection included receiving dialysis, taking immunosuppressive or anticancer medications, and having an underlying medical condition, such as diabetes, heart disease, and respiratory disease, such as chronic obstructive pulmonary disease (COPD).

Depression: Depression was assessed by the Japanese version of the Patient Health Questionnaire-9 (PHQ-9), which has been validated for assessing depression in primary care [34, 35]. The questionnaire is composed of 9 items scored on a four-point scale (0 to 3). A participant’s score can range from 0 to 27, with higher scores indicating more depressive symptoms, and a cut-off point ≥10 was a sensitive indicator of probable depression [34]. Although the cut-off point of the PHQ-9 indicates the possibility that a cut-off 11 point ≥11 may be suitable for the general population [35–37], the present study used the original cut-off point ≥10 to compare with the prevalence of probable depression in previous studies [33].

Anger: This study assessed the state anger scale and anger control scale, which are subscales of the State - Trait Anger Expression Inventory (STAXI) [38], which in Japanese was validated by Suzuki and Haruki [39]. The participants were asked “how do you feel about the social dislocations resulting from the COVID-19 pandemic” for the state anger scale and “what do you do when you are angry with the social dislocations” for the anger control scale, although originally the questions were “how do you feel now” and “what do you do when you get angry in your usual situation”. The state anger scale consists of 10 items scored on a four-point scale (0 to 3); a participant’s score can range from 0 to 27, and higher scores indicate higher state anger. The anger control scale consists of 7 items scored on a four-point scale (0 to 3); a participant’s score can range from 0 to 21, and higher scores indicate that the participant make a greater attempt to keep calm and restrain one’s behaviour.

Coping strategies: Coping strategies were assessed by the Japanese version of the Brief Coping Orientation to Problems Experienced (Brief COPE), which was validated by Otsuka et al. [40]. Although an original questions in the scale is “how do you feel and deal with troubling unpleasant things in daily life”, the present study asked “how do you feel and deal with social dislocations resulting from COVID-19 infection” to focus on the current situation. The scale comprises 28 items and assesses 14 coping styles: self-distraction, active coping, denial, substance use, use of emotional support, use of instrumental support, behavioural disengagement, venting, positive reframing, planning, humour, acceptance, religion, and self-blame. Each coping style was evaluated by two items scored on a four-point scale (1 to 4), and the scores for each coping style could range from 2 to 8. Higher scores indicated higher levels of coping styles.

### Statistical analysis

We calculated descriptive statistics, frequency distributions and means for all demographics and PHQ-9, state anger, anger control, and Brief COPE subscale scores. Based on the cut-off score of the PHQ-9, the participants were divided into a no-depression group (≤ 9) and a probable depression group (≥ 10). To verify differences between the no-depression group and the probable depression group regarding demographics and scores on measures, chi-squared tests, Student’s t-tests, and Welch tests were conducted. When regarding the Student’s t-tests and the Welch’s test, a Cohen’s d value was calculated. To reveal factors that affected probable depression, logistic regression analysis was carried out including all demographics, state anger score, anger control score, and all subscale scores of Brief COPE. The significance level was obtained with a *p*-value < .05 and confidence interval (CI) of 95%. A Cohen’s d value was calculated by the use of the G*Power program [41], and all of the other statistical analyses were performed using IBM SPSS Statistics Version 22.0. (Armonk, NY: IBM Corp.).

## Results

### Demographic variables and scores of the various measures

Participant demographic variables and scores on the various measures are presented in Additional Table 1. Although, we planned to recruit 2700 participants, a total of 2708 participants were included, with a mean age of 49.16 years (standard deviation (SD) = 16.32). Nearly the same number of individuals were in each age group (20s - 60s, all *N* = 454, 16.8%; 70s, *N* = 438, 16.2%), the same number of males and females were included (both *N* = 1354, 50%), and nearly the same number of individuals were included in each employment status category (full-rime worker, *N* = 956, 35.3%; no regular employment, *N* = 876, 32.4%; unemployed including homemaker, retired, and jobless, *N* = 876, 32.4%) since the survey used a quota sampling method to compare equal numbers across age groups, sexes, and employment statuses.

Based on the cut-off score of the PHQ-9, the number of patients in the no-depression group was 2211 (81.65%), and the number of patients in the probable depression group was 497 (18.35%). The probable depression group was younger (t = 12.91, df = 800.41, *p* < 0.001, d = 0.62), with a greater percentage of individuals in their 20s and 30s and a lower percentage of individuals in their 60s and 70s (*χ*^2^ = 146.65, *p* < 0.001), and included a greater percentage of males (*χ*^2^ = 10.41, *p* = 0.001), a lower percentage of full-time workers and homemakers and a greater percentage of individuals not working (*χ*^2^ = 55.79, *p* < 0.001), a greater percentage of participants with underlying disease (*χ*^2^ = 4.95, *p* = 0.026), a greater percentage of individuals who were single (*χ*^2^ = 129.1, *p* < 0.001), a greater percentage of those with less than 2 million JPY household income and a lower percentage of those with more than 8 million JPY (*χ*^2^ = 44.84, *p* < 0.001), and a lower percentage of participants who experienced no economic impact and a greater percentage of participants who experienced a negative economic impact (*χ*^2^ = 29.68, *p* < 0.001) than the no-depression group.

Regarding scores on measures, the probable depression group had higher scores on the PHQ-9 (t = 57.83, df = 594.89, *p* < 0.001, d = 3.21), state anger (t = 17.21, df = 588.98, *p* < 0.001, d = 0.96), self-distraction (t = 5.25, df = 2706, *p* < 0.001, d = 0.26), denial (t = 3.74, df = 651.29, *p* < 0.001, d = 0.20), substance use (t = 7.21, df = 636.19, *p* < 0.001, d = 0.39), use of emotional support (t = 2.74, df = 681.25, *p* < 0.01, d = 0.15), use of instrumental support (t = 2.74, df = 677.90, *p* = 0.006, d = 0.08), behavioural disengagement (t = 12.58, df = 660.97, *p* < 0.001, d = 0.65), venting (t = 5.21, df = 660.86, *p* < 0.001, d = 0.27), religion (t = 6.26, df = 655.67, *p* < 0.001, d = 0.33), and self-blame (t = 14.19, df = 628.77, *p* < 0.001, d = 0.76) and lower scores on active coping (t = 3.74, df = 2706, *p* < 0.001, d = 0.18) and planning (t = 2.42, df = 695.27, *p* = 0.016, d = 0.13) than the no-depression group.

### Relationship between probable depression and demographics, anger, and coping strategies

The results of multiple logistic regression with J-PHQ-9 as the objective variables are shown in Additional Table 2. Demographics with ORs greater than 1 were having an underlying disease (OR = 1.96, 95% CI = 1.32–2.92, *p* < 0.001), not working (OR = 1.85, 95% CI = 1.22–2.80, *p* < 0.01), and experiencing a negative economic impact (OR = 1.33, 95% CI = 1.01–1.77, *p* < 0.05), and demographics with ORs less than 1 were increased age (OR = 0.97, 95% CI = 0.96–0.98, *p* < 0.001), being married (OR = 0.53, 95% CI = 0.38–0.74, *p* < 0.001), and household income with more than 8 million JPY (OR = 0.45, 95% CI = 0.25–0.80). Both state anger and anger control had ORs greater than 1 (state anger: OR = 1.17, 95% CI = 1.14–1.21, p < 0.001; anger control: OR = 1.08, 95% CI = 1.04–1.13, *p* < 0.001). Regarding coping strategies, the strategies with ORs greater than 1 were behavioural disengagement (OR = 1.28, 95% CI = 1.13–1.44, *p* < 0.001) and self-blame (OR = 1.47, 95% CI = 1.31–1.65, *p* < 0.001), and the strategies with ORs less than 1 were denial (OR = 0.88, 95% CI = 0.77–0.99, *p* < 0.05), use of instrumental support (OR = 0.85, 95% CI = 0.76–0.95, *p* < 0.01), and planning (OR = 0.84, 95% CI = 0.74–0.94, *p* < 0.01). The OR for humour was less than 1 (OR = 0.89, *p* < 0.05); however, the CI level included 1 (95% CI 0.80–1.00).

## Discussion

In the context of the prolonged psychological distress resulting from the COVID-19 pandemic, the present study aimed to reveal the mental health of the general population during the second wave of infection in Japan and suggest concrete coping strategies.

### The prevalence of probable depression during the second wave of COVID-19

The present study showed that 18.35% of participants might have probable depression. Before the COVID-19 pandemic, the prevalence of depression in Japan was 2 to 8% [33, 42–44], which was similar to that reported the United States, i.e., a 7% prevalence [45]. The rates of probable depression during the second wave of COVID-19 was two to nine times higher than before the COVID-19 pandemic. Additionally, when the Great East Japan Earthquake occurred in 2011 in Japan, 12.18 to 14% of the general population who lived in Fukushima was depressive [46, 47]. Fukushima was the most damaged area by the earthquake. Accordingly, the mental health of the general population during the second wave of COVID-19 might be worse than mental health among Fukushima residents following the Great East Japan Earthquake.

The probable depression rates during this second wave in Japan might be the same as the rates in not only no-lockdown countries but also lockdown countries. Regarding no-lockdown areas, the probable depression rates during the first infection was reported to be 19% in Hong Kong and 23.6% in Turkey [9, 10]. On the other hand, regarding lockdown countries, some studies have reported high rates of probable depression, such as 48.3% in China and 41% in Spain [6, 17]; however, other studies have reported that 17.6 and 19.21% of the Chinese general population and 18.7% of the Spanish population had probable depression [3, 18, 48]. These rates of probable depression in lockdown countries were approximately the same as the rate in the present study. It was thought that mental health among the general population during the second wave of COVID-19 was going to be as bad or worse than the first wave of infection, although it is necessary to verify the rates of depression during the second wave in other studies and the depression rate among the Japanese general population during the first wave. Therefore, support for high-risk individuals who tend to have worse mental health and need coping strategies for maintaining their mental health will become even more important.

### Risk factors for increased incidences of probable depression symptoms

The results showed that the factors contributing to a high risk for increased depressive symptoms were, in order of magnitude, having an underlying disease, not working, having a low household income, being single, experiencing a negative economic impact by COVID-19, and being younger.

The individuals with an underlying disease that had a higher risk of more severe COVID-19, such as those receiving dialysis, taking immunosuppressive or anticancer medications, and having an underlying medical condition, such as diabetes, heart disease, and respiratory disease, such as COPD, had a 1.96-fold higher prevalence of probable depression than individuals without the underlying disease. The American Psychiatric Association [45] has pointed out that every illness was a risk factor for probable depression, and previous studies that investigated mental health during SARS-CoV-2 infection reported that prior psychiatric illness, physical illness, and chronic disease were strongly related to poor mental health [3, 10, 13, 15, 19]. The present study showed that underlying disease, which was associated with a higher risk of more severe COVID-19, was also strongly related to poor mental health. Additionally, it was thought that individuals with underlying diseases have to take preventive measures against COVID-19 because they had a higher risk of more severe COVID-19, and they might be more nervous than individuals without the underlying disease.

During the COVID-19 pandemic, the result that unemployment contributed to high risk was reported by a study [19]; however, two other studies reported that employment status, especially working outside the home, led to more depression than being unemployed [8, 49], and one other study reported that there were no significant associations between mental health and employment status [14]. These differences might have occurred based on the classification of employment status. Previous studies distinguished employment between working from home and working outside the home, while the present study distinguished between homemaker and not working. As this study has shown, homemakers might not be at high risk for poor mental health, while not working led to more depression than other statuses; note that the present study did not differentiate not working into retired and jobless.

Having a lower household income, experiencing a negative economic impact, and being unmarried as high risks were in agreement with previous studies [3, 11, 21–24]. We will focus on the prevalence of individuals experiencing a negative economic impact; 42.8% of our participants reported that they have experienced a negative economic impact because of COVID-19. In Cyprus, 48% of the general population reported experiencing a negative economic impact in April when it was 2 weeks into the lockdown [19]. The prevalence in Cyprus during lockdown was nearly the same as that in the present study during the second wave, which implied that even though Japan was not a lockdown country, the prevalence of experiencing an economic impact was the same as that in a lockdown country, although the severity of the economic impact is unclear.

Regarding the relationship between age and mental health during the COVID-19 pandemic, two sets of results have been reported by previous studies. One set of results showed that younger age was associated with a high risk for psychological distress [4, 11, 13, 17, 19, 21, 22], and another set of results showed that older age was associated with a high risk [3, 50]; the results in the present study were consistent with the former set of studies. The former set of results was thought to indicate that older adults could deal with stressors in a more adaptive way than younger adults [11, 51], and the latter set of results was thought to indicate that elderly adults reported more psychological stress because many elderly individuals had chronic disease [3]. Since the present study involved an online survey, the older participants had good enough eyesight and cognitive function to use a device such as a mobile phone or personal computer. Accordingly, for the elderly individuals with chronic disease and for younger adults, it is necessary to inform them about adaptive strategies and provide medical treatment.

### Relationship between probable depression and anger and effective coping strategies for probable depression during the second wave of COVID-19

The present study showed that state anger over social dislocations resulting from the COVID-19 pandemic and an attempt to control anger increased probable depression, although the associations were weak. State anger was associated with probable depression because anger is a psychological stress response similar to depression, anxiety, displeasure, and apathy [52]. On the other hand, anger control means suppressing one’s anger by attempting to keep calm and restraining one’s behaviour, and anger control is considered positive psychological functioning [53]. It was expected that anger control decreases probable depression. There is a hypothesis that controlling anger causes an increase in anger such that suppressing anger has been linked to anger rumination [54, 55]. The results of the present study suggested that suppressing anger and the link to anger rumination might cause probable depression because of its link to anger rumination; accordingly, forgetting anger is better than suppressing anger in the context of social dislocations.

The present study suggested that the use of instrumental support, planning, and denial were effective coping strategies, and behavioural disengagement and self-blame were ineffective coping strategies. The results regarding denial are discussed below, and we will discuss the other coping strategies here. Instrumental support and planning have been classified as problem-focused coping strategies, while behavioural disengagement and self-blame have been classified as dysfunctional coping strategies [56]. Indeed, previous studies that investigated occupational stress and problems in one’s daily lives reported the same results [40, 57]. Accordingly, despite the social dislocations resulting from the COVID-19 pandemic, effective and ineffective coping strategies were the same as usual: it is recommended to get help and advice from other people about what to do and to try to come up with a strategy about what to do, while it is not recommended to give up trying to deal with the social dislocations and to criticize and blame oneself.

Regarding denial, because it is classified among the dysfunctional coping strategies such as behavioural disengagement and self-blame [56], using denial has been associated with probable depression, low levels of concentration, and low activity levels [40, 58, 59]. However, when an individual cannot cope with the stressor by oneself and the stressor is prolonged, using denial might be an effective coping strategy [60]. Because the social dislocations resulting from the COVID-19 pandemic cannot be resolved by individuals, and there are no signs that social dislocations are being brought under control, keeping away from social dislocations might be useful for maintaining mental health.

Although denial might prevent probable depression during the pandemic, the probable depression group used denial much more than the no-depression group. The influence of denial was smaller than other risk factors, such as having an underlying disease, a particular employment status, and a particular household income. Additionally, the probable depression group used many more ineffective coping strategies than the no-depression group, that is, self-blame and behavioural disengagement. The influence of these ineffective coping strategies was larger than the influence of denial. Consequently, it is thought that the probable depression group used much denial, which might be an effective coping strategy for depression during the second wave of COVID-19; however, the influence of denial was smaller than the risk factors and ineffective coping strategies, so depression could not be prevented.

### Limitations

The present study has some limitations. First, because the present study aimed to reveal risk factors and coping strategies against long-term social dislocations, we recruited participants who lived in the 13 prefectures under special precautions during the COVID-19 pandemic. Accordingly, the prevalence of probable depression in this study might be higher than in other than the 13 prefectures. Second, due to the fact that a web-based survey may be the safest and most suitable method in situations of a COVID-19 infection, the participants were recruited by a web-based survey; thus, random sampling could not be conducted, and these results cannot reflect the prevalence of the entire Japanese population. Third, the present study may indicate probable depression, but the present study cannot explicitly determine that actual depression occurred. Fourth, this study could not assess other mental health factors, such as anxiety and the quality of life. To determine the local situation in Japan, a longitudinal survey, as well as several surveys, are needed. Finally, as responses to the survey were self-reported, emotions and behaviours among participants have not been observed, especially the state anger, anger control, and coping strategies.

## Conclusions

The present study showed that 1 out of 5 individuals in the general population might have experienced probable depression during the second wave of COVID-19, even though they were not in a lockdown countries. Mental health might be equivalent to or worse than mental health during the first infection, which was thought to be caused by long-term social dislocations. Adaptive coping strategies need to be made known to the public and support for high-risk individuals is needed; this is especially true for depressive individuals, who tend to give up trying to deal with social dislocations and criticize and blame themselves. Depressive individuals used many more coping strategies than no-depressive individuals; however, many coping strategies that depressive individuals used were ineffective coping strategies. It is recommended that individuals obtain help and advice from other people about what to do, try to develop a strategy about what to do, and keeping away from the social dislocations might be an adaptive strategy in this situation. Although some coping strategies are useful for maintaining mental health during COVID-19, demographics, such as marital status and employment status, had more powerful effects on mental health than these coping strategies. Individuals who had underlying disease, were not working, had low household income, were single, experienced a negative economic impact by COVID-19, and were younger tended to be depressive, and they need to take medical treatment because there is a limit to the effort that an individual can make.

## Supplementary Information

**Additional file 1: Table S1.** Participant demographic characteristics, information related to social dislocations resulting from the COVID-19 pandemic, and scores from the PHQ-9, State Anger, Anger Control, and Brief COPE.

**Additional file 2: Table S2.** Multivariate logistic regression analysis of probable depression by set variables.

## Data Availability

The datasets generated during the current study are available in the openICPSR database, 10.3886/E121361V1.

## Abbreviations

SD: Standard deviation
COVID-19: Coronavirus infection
PHQ-9: Patient Health Questionnaire-9
OR: Odds ratio
CI: Confidence interval
COPD: Chronic obstructive pulmonary disease
STAXI: State - Trait Anger Expression Inventory
Brief COPE: Brief Coping Orientation to Problems Experienced
