# Supplementary material for: Depression, risk factors, and coping strategies in the context of social dislocations resulting from the second wave of COVID-19 in Japan
Source: BMC Psychiatry. 2021 Jan 12;21:33. doi: 10.1186/s12888-021-03047-y (PMC7802816; doi:10.1186/s12888-021-03047-y)
Supplement: Supplementary file 1 — Additional file 1: Table S1. Participant demographic characteristics, information related to social dislocations resulting from the COVID-19 pandemic, and scores from the PHQ-9, State Anger, Anger Control, and Brief COPE. [file 12888_2021_3047_MOESM1_ESM.docx]

**Depression, risk factors, and coping strategies in the context of social dislocations resulting from the second wave of COVID-19 in Japan**

Yuko Fukase, Kanako Ichikura, Hanako Murase, and Hirokuni Tagaya

**Additional Table 1.** Participant demographic characteristics, information related to social dislocations resulting from the COVID-19 pandemic, and scores from the PHQ-9, State Anger, Anger Control, and Brief COPE.

|  | All participants  N = 2,708 | | No-depression  N = 2,211 | | Probable depression  N = 497 | | *χ*^2^ or *t* |
| --- | --- | --- | --- | --- | --- | --- | --- |
|  | N or Mean | % or SD | N or Mean | % or SD | N or Mean | % or SD |  |
| **Demographics** |  |  |  |  |  |  |  |
| Age, Mean **±** SD | 49.16 | 16.32 | 50.91 | 16.21 | 41.41 | 14.48 | 12.91*** |
| Age group, N, % |  |  |  |  |  |  | 146.65*** |
| 20s | 454 | 16.8 | 311 | 14.1 | 143 | 28.8 |  |
| 30s | 454 | 16.8 | 343 | 15.5 | 111 | 22.3 |  |
| 40s | 454 | 16.8 | 358 | 16.2 | 96 | 19.3 |  |

**Additional Table 1 continued**

|  | All participants | | No-depression | | Probable depression | | *χ*^2^ or *t* |
| --- | --- | --- | --- | --- | --- | --- | --- |
| 50s | 454 | 16.8 | 367 | 16.6 | 87 | 17.5 |  |
| 60s | 454 | 16.8 | 423 | 19.1 | 31 | 6.2 |  |
| 70s | 438 | 16.2 | 409 | 18.5 | 29 | 5.8 |  |
| Sex, N, % |  |  |  |  |  |  | 10.41** |
| Male | 1354 | 50.0 | 1073 | 48.5 | 281 | 56.5 |  |
| Female | 1354 | 50.0 | 1138 | 51.5 | 216 | 43.5 |  |
| Employment status, N, % | |  |  |  |  |  | 55.79*** |
| Full-rime worker | 956 | 35.3 | 821 | 37.1 | 135 | 27.2 |  |
| No regular employment | 876 | 32.4 | 705 | 31.9 | 171 | 34.4 |  |
| Homemaker | 392 | 14.5 | 341 | 15.4 | 51 | 10.3 |  |
| Not working | 484 | 17.9 | 344 | 15.6 | 140 | 28.2 |  |

**Additional Table 1 continued**

|  | All participants | | No-depression | | Probable depression | | *χ*^2^ or *t* |
| --- | --- | --- | --- | --- | --- | --- | --- |
| Residential areas, N, % |  |  |  |  |  |  |  |
| Hokkaido | 193 | 7.1 | 150 | 6.8 | 43 | 8.7 |  |
| Ibaraki | 66 | 2.5 | 53 | 2.4 | 13 | 2.6 |  |
| Saitama | 277 | 10.2 | 234 | 10.6 | 43 | 8.7 |  |
| Chiba | 220 | 8.1 | 176 | 8.0 | 44 | 8.9 |  |
| Tokyo | 507 | 18.7 | 417 | 18.9 | 90 | 18.1 |  |
| Kanagawa | 325 | 12.0 | 272 | 12.3 | 53 | 10.7 |  |
| Ishikawa | 42 | 1.6 | 33 | 1.5 | 9 | 1.8 |  |
| Gifu | 58 | 2.1 | 47 | 2.1 | 11 | 2.2 |  |
| Aichi | 267 | 9.9 | 218 | 9.9 | 49 | 9.9 |  |
| Kyoto | 91 | 3.4 | 72 | 3.3 | 19 | 3.8 |  |
| Osaka | 328 | 12.1 | 270 | 12.2 | 58 | 11.7 |  |

**Additional Table 1 continued**

|  | All participants | | No-depression | | Probable depression | | *χ*^2^ or *t* |
| --- | --- | --- | --- | --- | --- | --- | --- |
| Hyogo | 205 | 7.6 | 169 | 7.6 | 36 | 7.2 |  |
| Fukuoka | 129 | 4.8 | 100 | 4.5 | 29 | 5.8 |  |
| Underlying disease, N, % | |  |  |  |  |  | 4.95* |
| Without | 2389 | 88.2 | 1965 | 88.9 | 424 | 85.3 |  |
| With | 319 | 11.8 | 246 | 11.1 | 73 | 14.7 |  |
| Marital status, N, % |  |  |  |  |  |  | 129.1*** |
| Single | 1216 | 44.9 | 879 | 39.8 | 337 | 67.8 |  |
| Married | 1492 | 55.1 | 1332 | 60.2 | 160 | 32.2 |  |
| Household income, N, % | |  |  |  |  |  | 44.84*** |
| < 2 million JPY | 299 | 11.0 | 214 | 9.7 | 85 | 17.1 |  |
| 2 - 8 million JPY | 1434 | 53.0 | 1182 | 53.5 | 252 | 50.7 |  |
| > 8 million JPY | 389 | 14.4 | 352 | 15.9 | 37 | 7.4 |  |
| I do not know | 297 | 11.0 | 230 | 10.4 | 67 | 13.5 |  |

**Additional Table 1 continued**

|  | All participants | | No-depression | | Probable depression | | *χ*^2^ or *t* |
| --- | --- | --- | --- | --- | --- | --- | --- |
| Non-response | 289 | 10.7 | 233 | 10.5 | 56 | 11.3 |  |
| Economically impacted, N, % | |  |  |  |  |  | 29.68*** |
| Without impact | 1471 | 54.3 | 1254 | 56.7 | 217 | 43.7 |  |
| Negative impact | 1160 | 42.8 | 893 | 40.4 | 267 | 53.7 |  |
| Positive impact | 77 | 2.8 | 64 | 2.9 | 13 | 2.6 |  |
| **Scores on measures** |  |  |  |  |  |  |  |
| PHQ-9, mean **±** SD | 5.30 | 5.41 | 3.19 | 2.76 | 14.66 | 4.22 | 57.83*** |
| State Anger, mean **±** SD | 15.18 | 5.38 | 14.16 | 4.37 | 19.71 | 6.89 | 17.21*** |
| Anger Control, mean **±** SD | 19.10 | 4.03 | 19.06 | 4.06 | 19.27 | 3.91 | 1.05 |
| Brief COPE, mean **±** SD | |  |  |  |  |  |  |
| Self-distraction | 4.70 | 1.37 | 4.63 | 1.34 | 4.99 | 1.43 | 5.25*** |
| Active coping | 5.26 | 1.30 | 5.30 | 1.29 | 5.06 | 1.35 | 3.74*** |
| Denial | 3.15 | 1.24 | 3.10 | 1.18 | 3.36 | 1.45 | 3.74*** |

**Additional Table 1 continued**

|  | All participants | | No-depression | | Probable depression | | *χ*^2^ or *t* |
| --- | --- | --- | --- | --- | --- | --- | --- |
| Substance use | 3.20 | 1.54 | 3.08 | 1.44 | 3.72 | 1.85 | 7.21*** |
| Emotional support | 4.07 | 1.45 | 4.03 | 1.41 | 4.25 | 1.60 | 2.74** |
| Instrumental support | 4.06 | 1.48 | 4.04 | 1.44 | 4.17 | 1.65 | 1.60 |
| Behavioural  disengagement | 3.88 | 1.31 | 3.72 | 1.22 | 4.60 | 1.46 | 12.58*** |
| Venting | 4.06 | 1.35 | 3.99 | 1.29 | 4.37 | 1.54 | 5.21*** |
| Positive reframing | 4.75 | 1.42 | 4.76 | 1.40 | 4.68 | 1.51 | 1.02 |
| Planning | 5.07 | 1.39 | 5.10 | 1.37 | 4.92 | 1.49 | 2.42* |
| Humour | 3.68 | 1.36 | 3.67 | 1.31 | 3.73 | 1.55 | 0.77 |
| Acceptance | 5.97 | 1.32 | 5.99 | 1.31 | 5.91 | 1.36 | 1.14 |
| Religion | 3.25 | 1.38 | 3.16 | 1.31 | 3.64 | 1.59 | 6.26*** |
| Self-blame | 3.52 | 1.40 | 3.31 | 1.25 | 4.43 | 1.66 | 14.19*** |

Notes: * p < 0.05, ** p < 0.01, *** p < 0.001
