# Supplementary material for: Depression, risk factors, and coping strategies in the context of social dislocations resulting from the second wave of COVID-19 in Japan
Source: BMC Psychiatry. 2021 Jan 12;21:33. doi: 10.1186/s12888-021-03047-y (PMC7802816; doi:10.1186/s12888-021-03047-y)
Supplement: Supplementary file 2 — Additional file 2: Table S2. Multivariate logistic regression analysis of probable depression by set variables. [file 12888_2021_3047_MOESM2_ESM.docx]

**Additional Table 2.** Multivariate logistic regression analysis of probable depression by set variables.

| Predictor variable | | β | SE | Wald | OR | 95% CI |
| --- | --- | --- | --- | --- | --- | --- |
| Age |  | -0.03 | 0.00 | 38.61 | 0.97 | 0.96 - 0.98*** |
| Underlying disease | Without (reference) |  |  |  |  |  |
|  | With | 0.67 | 0.20 | 11.07 | 1.96 | 1.32 - 2.92*** |
| Marital status | Single (reference) |  |  |  |  |  |
|  | Married | -0.64 | 0.17 | 14.10 | 0.53 | 0.38 - 0.74*** |
| Employment status | Regular employee (reference) |  |  |  |  |  |
|  | No regular employment | 0.04 | 0.18 | 0.04 | 1.04 | 0.73 - 1.47 |
|  | Homemaker | 0.20 | 0.26 | 0.58 | 1.22 | 0.74 - 2.01 |
|  | Not working | 0.62 | 0.21 | 8.49 | 1.85 | 1.22 - 2.80** |
| Household income | < 2 million JPY (reference) |  |  |  |  |  |
|  | 2 - 8 million JPY | -0.13 | 0.19 | 0.48 | 0.87 | 0.60 - 1.28 |
|  | > 8 million JPY | -0.80 | 0.29 | 7.46 | 0.45 | 0.25 - 0.80** |

**Additional Table 2 continued**

| Predictor variable | | β | SE | Wald | OR | 95 % CI |
| --- | --- | --- | --- | --- | --- | --- |
| Economic impact | Without impact (reference) |  |  |  |  |  |
|  | Negative impact | 0.29 | 0.14 | 4.01 | 1.33 | 1.01 - 1.77* |
|  | Positive impact | -0.53 | 0.45 | 1.41 | 0.59 | 0.25 - 1.41 |
| State Anger |  | 0.16 | 0.01 | 137.54 | 1.17 | 1.14 - 1.21*** |
| Anger Control |  | 0.08 | 0.02 | 13.17 | 1.08 | 1.04 - 1.13*** |
| Brief COPE | Denial | -0.13 | 0.07 | 4.19 | 0.88 | 0.77 - 0.99* |
|  | Use of instrumental support | -0.17 | 0.06 | 8.41 | 0.85 | 0.76 - 0.95** |
|  | Behavioural disengagement | 0.25 | 0.06 | 15.90 | 1.28 | 1.13 - 1.44*** |
|  | Planning | -0.18 | 0.06 | 8.62 | 0.84 | 0.74 - 0.94** |
|  | Humour | -0.11 | 0.06 | 3.84 | 0.89 | 0.80 – 1.00* |
|  | Self-blame | 0.38 | 0.06 | 41.76 | 1.47 | 1.31 - 1.65*** |

Notes: * p < 0.05, ** p < 0.01, *** p < 0.001; SE: standard error; OR: odds ratio; 95 % CI: confidence interval at the 95 % level.
